# Liver fibrosis and retinal features in an older Mediterranean population: Results from the Salus in Apulia study

**DOI:** 10.3389/fnins.2022.1048375

**Published:** 2022-12-15

**Authors:** Luisa Lampignano, Alfredo Niro, Fabio Castellana, Ilaria Bortone, Roberta Zupo, Sarah Tirelli, Rossella Tatoli, Chiara Griseta, Sara De Nucci, Annamaria Sila, Giovanni De Pergola, Caterina Conte, Giovanni Alessio, Francesco Boscia, Giancarlo Sborgia, Eye Clinic Research Group, Giulia Maria Pia Bisceglia, Gianluigi Giannelli, Rodolfo Sardone

**Affiliations:** ^1^Unit of Research Methodology and Data Sciences for Population Health, “Salus in Apulia Study”, National Institute of Gastroenterology Istituto di Ricovero e Cura a Carattere Scientifico (IRCCS) “Saverio de Bellis”, Research Hospital, Castellana Grotte, Italy; ^2^Eye Clinic, Hospital “SS. Annunziata”, Azienda Sanitaria Locale (ASL) Taranto, Taranto, Italy; ^3^Unit of Geriatrics and Internal Medicine, National Institute of Gastroenterology “Saverio de Bellis”, Research Hospital, Bari, Italy; ^4^Department of Human Sciences and Promotion of the Quality of Life, San Raffaele Roma Open University, Rome, Italy; ^5^Department of Endocrinology, Nutrition and Metabolic Diseases, IRCCS MultiMedica, Milan, Italy; ^6^Department of Basic Medical Sciences, Neuroscience and Sense Organs, University of Bari Aldo Moro, Bari, Italy; ^7^Scientific Direction, National Institute of Gastroenterology IRCCS “Saverio de Bellis”, Research Hospital, Castellana Grotte, Italy

**Keywords:** liver, fibrosis, retina, older population, aging

## Abstract

**Background:**

Age is a leading contributor to the liver fibrosis rate and a gradual deterioration of optical function, but this association in older populations is still under-explored. The present study aimed to explore the link between vascular and neural retinal characteristics and the risk of liver fibrosis in 731 older adults from the population-based Salus in Apulia study.

**Methods:**

Retinal features were obtained using optical coherence tomography (OCT) and OCT-angiography (OCT-A). Liver fibrosis risk was taken as the fibrosis-4 (FIB-4) score. Generalized linear models (logistic regression) were used to estimate the association effect between each unit increase of OCT and OCT-A parameters as independent variables and a FIB-4 ≥ 2.67 score as an outcome. Generalized additive models were used to assess the non-linear association between OCT-A features and the linear FIB-4 score.

**Results:**

Increased gangliar cell complex (GCC) thickness was inversely associated with a FIB-4 score above the cut-off in both the raw model (OR: 0.98; 95% CI: 0.96–0.99; SE: 0.01) and after adjustment for age, sex, education, hypertension, diabetes, total cholesterol, and triglycerides (OR: 0.98; 95% CI: 0.97–0.99; SE: 0.01).

**Conclusion:**

Our findings add to the growing volume of scientific literature demonstrating that liver fibrosis is associated with retinal neurodegeneration. This study raises a number of new questions, including whether OCT-A may be used to track the progression of metabolic abnormalities and define exact thresholds for predicting and classifying liver disease.

## Introduction

All chronic liver conditions exist on a gradient, with hepatic fibrosis acting as a forerunner to end-stage cirrhosis (Altamirano-Barrera et al., 2017). Subclinical liver fibrosis affects up to 15% of people who have no clinically visible signs, symptoms, or laboratory proof of liver disease (Caballería et al., 2018). Average liver enzyme values are found in the majority of patients with subclinical but substantial liver fibrosis (Koehler et al., 2016), prompting the need for novel biomarkers to identify affected individuals.

It has been suggested that during the aging process, the susceptibility to liver fibrosis increases (Kim et al., 2015). One of the biological mechanisms that could explain this predisposition is increased oxidative stress and reduced tolerance to oxidative damage (Kim et al., 2015). At the molecular and cellular levels, metabolic changes are inextricably linked to aging and age-related disease (Feng et al., 2016; Dhillon and Denu, 2017).

As well as the liver parenchyma, the retina is one of the most metabolically active tissues (Fort et al., 2014) and mitochondrial dysfunction has been described in the aged retina and optic nerve, with a decreased energy metabolism and impaired antioxidant defenses (Kubota et al., 2016; Ferrington et al., 2017). These mechanisms underlie, at least in part, the age-related structural changes in the ocular tissues, particularly the retina and optic nerve, that cause chronic visual impairment (Gambato et al., 2015).

Recently, the importance of the biochemical interplay between neural cells and blood vessels, and its dysfunction due to aging and aging-related diseases (Cai et al., 2017) was highlighted, including advanced liver disease (Claeys et al., 2021). To better understand the aging process and determine novel targets for disease treatment, detecting metabolic changes that occur in the aging eye could help to pinpoint deterioration levels (Wang et al., 2018).

Retinal findings offer an opportunity to non-invasively explore systemic health in the human body. In addition, retinal vasculature has been linked to systemic microvascular health, and alterations have been linked to metabolic syndrome (Kawasaki et al., 2008), increased cardiovascular risk (Farrah et al., 2019, 2020; Allon et al., 2021), including the incidence of stroke (Wong et al., 2001) and coronary heart disease (Daien et al., 2013), cognitive decline and brain changes related to aging and vascular disease (Wong et al., 2002; Baker et al., 2007; Ding et al., 2008; Heringa et al., 2013; Czakó et al., 2020). In particular, high-resolution retinal B-scan images using optical coherence tomography (OCT) technology previously confirmed thinning of the neuroretinal layers in patients with liver cirrhosis as compared to age-matched control subjects (Gifford et al., 2020).

Specifically, OCT-angiography (OCT-A), an innovative extension of OCT technology that provides non-invasive depth-resolved visualization of the retinal microvasculature (Spaide et al., 2015; Kashani et al., 2017), has been successfully used to explore the predictive power of retinal vessel features for detecting cardiovascular diseases (Monteiro-Henriques et al., 2022), systemic hypertension (Takayama et al., 2018; Niro et al., 2022), neurodegenerative disorders (Czakó et al., 2020; Pellegrini et al., 2020; Pinhas et al., 2020), diabetes mellitus (Le et al., 2021), and chronic kidney disease (Vadalà et al., 2019).

This cross-sectional study aimed to investigate the association between vascular and neural retinal features and the risk of liver fibrosis in an aging population from a Mediterranean area.

## Materials and methods

### Study population and design

Data used in the present cross-sectional study were drawn from the population-based Salus in Apulia Study conducted on subjects over 65 years, residents in Castellana Grotte, Bari (Puglia Region, Southern Italy). The sampling frame was the 19,675 residents listed in the health registry office on 31 December 2014, 4,021 of whom were aged 65 years or older. The study design and data collection methods have been described in detail elsewhere (Lozupone et al., 2018). This study presents data from a subpopulation that underwent OCT and OCT-A (*n* = 731). Moreover, we excluded people who developed cirrhosis or advanced fibrosis at earlier points in life. All participants signed informed consent, and the study was approved in 2014 and again in 2019 by the IRB of the National Institute of Gastroenterology “S. De Bellis,” where all the examinations described in this study were performed. The present study adhered to the “Standards for Reporting Diagnostic Accuracy Studies” (STARD) guidelines,^1^ the “Strengthening the Reporting of Observational Studies in Epidemiology” (STROBE) guidelines,^2^ and is following the Helsinki Declaration of 1975.

### Clinical and lifestyle assessment

Body mass index (BMI) was calculated as kg/m^2^. Height and weight measurements were performed using a Seca 220 stadiometer and a Seca 711 scale. Blood samples were collected in the morning after overnight fasting to measure the levels of fasting blood glucose (FBG), glycated hemoglobin (HbA1c), total cholesterol, transaminases, and triglycerides, using standard automated enzymatic colorimetric methods (AutoMate 2550; Beckmann Coulter, Brea, CA, USA), under strict quality control. The platelet count was determined by a Coulter Hematology analyzer (Beckman Coulter). The clinical evaluation included extemporaneous ambulatory systolic blood pressure (SBP) and diastolic blood pressure (DBP), determined in a sitting position after at least a 10-min rest, and at least three times, using the OMRON M6 automatic blood pressure monitor. According to the American Heart Association criteria, hypertension was diagnosed when systolic pressure was ≥130 mmHg or diastolic pressure ≥80 mmHg (Whelton et al., 2018). Diabetes mellitus was diagnosed as FBG ≥ 126 mg/dl. Education was defined by years of schooling. Smoking status was assessed with the question, “Are you a current smoker?”

The level of education is expressed in years of schooling. Lastly, alcohol consumption was evaluated by a validated food frequency questionnaire (FFQ) (Leoci et al., 1933). Estimates of daily alcohol consumption were calculated from Italian food composition tables (Carnovale, 2022). According to European guidelines for daily alcohol consumption, a threshold of 20 g/day in women and 30 g/day in men was used (European Association for the Study of the Liver [EASL] et al., 2016).

### Fibrosis-4 index for liver fibrosis risk

Fibrosis-4 (FIB-4) is a non-invasive test that combines standard biochemical values (platelets, ALT, and AST) and age. FIB-4 can accurately exclude advanced fibrosis in NAFLD patients and thus provide a valid, reliable first-line measure of liver fibrosis for primary or secondary care (Shah et al., 2009; McPherson et al., 2010), even in subjects ≥65 years (McPherson et al., 2017). Simple non-invasive fibrosis scores such as FIB-4 are generally used to classify or exclude advanced fibrosis as part of a step-by-step approach to diagnosis and risk stratification in patients with NAFLD, as recommended in most guidelines (Ratziu et al., 2010; Chalasani et al., 2012).

The formula for FIB-4 is:


Age([yr]×AST[U/L])/((PLT[10(9)/L])×(ALT[U/L])(1/2)).

As suggested by McPherson and colleagues (McPherson et al., 2017), three age-specific cut-of points for subjects aged 65+ years were used to assess the liver fibrosis risk (low-risk: ≤2, intermediate-risk: 2–2.66, and high-risk: ≥2.67). In our study, only the high-risk (≥2.67) cut-off was applied given its proven effectiveness in improving specificity for advanced fibrosis.

### Ophthalmological assessment

**FIGURE 1:**
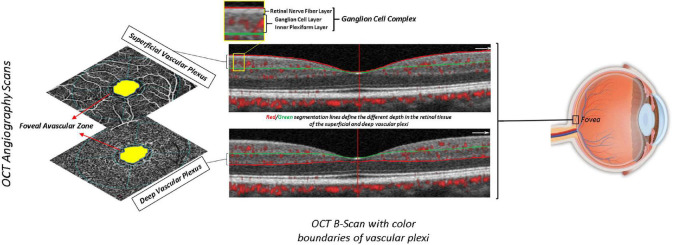
Retinal features from OCT-A scan **(A)** and OCT-B scan **(B)**.

Each participant underwent a complete ophthalmic examination including best-corrected visual acuity (BCVA) measurement, slit-lamp biomicroscopy, intraocular pressure (IOP) measurement, and funduscopy, as per the protocol of the Salus in Apulia Study (Lozupone et al., 2018). The evaluation of anatomical and functional parameters was useful to select the subjects to be included in the study, in accordance with ophthalmic exclusion criteria, as previously reported (Niro et al., 2022). BCVA was recorded as Snellen visual acuity and converted to the logarithm of minimal angle of resolution (LogMar) units for statistical analysis. Then, we performed OCT and OCT-A using Optovue RTVue XR 100 AVANTI, Optovue, Inc. OCT-A analyzes retinal vasculature after identifying and segmenting multiple retinal layers using the AngioVue module with Optovue RTVue AVANTI software (version 2015.100.0.35, Optovue, Inc., Fremont, CA, USA). The Angio Retina mode (3 mm × 3 mm) and the Angio Disc mode (4.5 mm × 4.5 mm) were employed. The software also provided the signal strength index (SSI), which represents the scan’s reflectance signal strength, and a quality index (*Q*-score), representing the overall quality of the image, taking into account factors like SSI and motion artifacts (Czakó et al., 2019). In the present study, we only included images with a *Q*-score of 6 or above, SSI above 60, and without motion or shadow artifacts. The examinations were performed blinded by three trained ophthalmologists. The vessel density (VD, %) was defined as the proportion of vessel area with blood flow over the total area measured and automatically measured by the OCT software. The OCT angiograms centered on the fovea automatically defined the superficial and deep vascular plexus. The VD at each plexus, superficial VD (SVD) and deep VD (DVD), was calculated for the whole 3-mm circle area centered on the fovea (whole retina) (Figure 1A). The thickness (μm) of the ganglion cell complex (GCC), consisting of the thickness of the retinal nerve fiber layer (RNFL), ganglion cell layer (GCL), and inner plexiform layer (IPL) (Figure 1B), at the macular area, and, separately, of the RNFL, was measured at the same time using the same OCT. The device measures GCC and RNFL thickness within an automatically rendered 7 mm^2^ area, centered 1 mm temporally to the fovea, and calculates the probabilities of deviation from the normal range of thickness based on comparison with an age-matched control group of healthy subjects (Niro et al., 2022). Each retinal feature shown in Figure 1 is explained in Table 1.

**TABLE 1:** Description of the retinal features shown in Figure 1.

		Retinal features	Description	Legend
OCT angiography scan	Vascular plexi	Superficial vascular plexus	The superficial vascular plexus was comprised between the inner limiting membrane (red line) and 9 micron above the junction between the inner plexiform layer and the inner nuclear layer (green line). Lower vascular density indicates a poor neuroretinal vascular perfusion.	a
		Deep vascular plexus	The deep vasculare plexus was comprised between 9 micron above the inner plexiform layer junction (green line) and 9 micron below the outer plexiform layer and outer nuclear layer junction (red line).	
		Foveal avascular zone	It is a region within the fovea centralis at the center of the retina of the human eye that is devoid of retinal blood vessels. The geometric center of the FAZ is taken to be the center of the macula and thus the point of fixation.	
OCT-B scan	Ganglion cellular complex	Retinal nerve fiber layer	Is neural tissue formed by the expansion of the fibers of the optic nerve that meet the retinal cells with citoneural junctions. The thickness of the RNFL decreases with age and pathological conditions of the inner retina and optic nerve, including neurodegeneration.	b
		Inner plexiform layer	This layer consists of synaptic connections between the axons of bipolar cells and dendrites of ganglion cells. To the OCT scan, the reduction of this layer is a pathological mark for the neuro-retina.	
		Ganglion cell layer	Is a neural tissue connected to the IPL and represents the inner neural layer of the retina. The reduction of GCL can be interpreted as a form of neural damage to the retina or optic nerve head, or more extensively as a neurodegeneration marker.	

Exclusion criteria for all study participants were IOP > 22 mmHg, history s were IOP > 22 mmHg, history of glaucoma, optic neuropathies, a recent history of intraocular surgery, ocular trauma, and an obvious media opacity that could interfere with the OCT analysis.

### Statistical analysis

Continuous variables were expressed as mean ± standard deviation (SD), median (min to max) and categorical variables as the proportion (%). Statistical significance was set with an effect size. The whole sample was subdivided into two groups according to the FIB-4 cut-off (2.67). Due to the diffuse non-normal distribution of all variables (using the Shapiro distribution test), Wilcoxon effect size was performed to assess the magnitude of differences between the groups for continuous variables and prevalence difference for categorical variables. General linear logistic regression models were used to estimate the association effect between each unit increase of OCT-A parameters as independent variables and a FIB-4 ≥ 2.67 score as an outcome. The retinal features were based on of significant differences in group (Fibrosis Risk vs. non-Fibrosis Risk) comparisons (Table 1). To assess the confounding effect of several covariates, we built two hierarchical nested general linear logistic models: an unadjusted and a fully adjusted model. The covariates were age, sex, education, hypertension, diabetes, alcohol consumption, and serum levels of total cholesterol and triglycerides. They were selected for their probable confounding effect associated with retinal functions and liver health (Koehler et al., 2016; Zupo et al., 2021; Niro et al., 2022). In addition to the logistic models, we implemented an interpretable model that explains class scores (the logit of class probabilities) using a sum of univariate and bivariate shape functions of predictors: the generalized additive model (GAM). The GAM was used in order to able non-linear associations between the predictors and outcomes that general models cannot fit. The Regression Function is modified in GAMs, and only due to this transformation do the GAMs work better in terms of Generalization to random unseen data, fitting the data very smoothly and flexibly, mostly without adding Complexity or much variance to the Model. The basic idea in Splines is fit Smooth Non-linear Functions on a bunch of Predictors Xi to capture and learn the Non-linear relationships between the Model variables, i.e., X and Y. The term Additive expresses the process of fitting and retaining the additivity of the Linear Models. A graphic function will be used to show the probability curve of each covariate in the selected GAM model, to visualize the linearity or non-linearity of the relation. Sensitivity analysis was constructed by starting with a crude logistic regression model and adding each regressor individually until a totally correct model was obtained.

Moreover, to reduce selection bias due to the presence of a dominant eye in each participant and simplify the reading of results, we used a complete randomization algorithm for the eye selection, assigning the corresponding value (left or right eye) to the new variable thus created. Statistical analysis was performed with RStudio software, Version 1.4.1106, using additional packages: Tidyverse, randomizeR, rstatistix, Epi, kableExtra, and gmodels.

## Results

The participants average age was 73.4 ± 6.1 years, and there was higher percentage of females (*n* = 434, 59.4%). The main sociodemographic and clinical characteristics of the whole sample subdivided according to the FIB-4 cut-off (2.67) are shown in Table 2. People with higher FIB-4 were older [standard error (SE) −0.70 (−0.89 to −0.51)], and there was no significant difference in terms of BMI between the two groups.

**TABLE 2:** Description of the whole sample according to the FIB cut-offs.

	FIB < 2.67		FIB ≥ 2.67		
	Mean ± SD	Median (min to max)	Mean ± SD	Median (min to max)	Effect size*
Proportions (%)	587 (80.30)		144 (19.70)		
Age (years)	72.56 ± 5.76	71 (65–95)	76.66 ± 6.24	77 (65–91)	**−0.70 (−0.89 to −0.51)**
**Sex**					
Male	242 (41.20)		55 (38.20)		3.03 (−5.85 to 11.91)
Female	345 (58.80)		89 (61.80)		
Current smoker (yes)	35 (6.00)		10 (6.90)		0.98 (−3.59 to 5.55)
BMI (Kg/m^2^)	28.22 ± 4.66	27.97 (18.36–47.69)	27.85 ± 5.01	27.11 (19.53–44.3)	0.08 (−0.10 to 0.26)
DBP (mmHg)	78.33 ± 7.23	80 (50–100)	75.87 ± 8.97	80 (40–100)	**0.27 (0.10–0.45)**
SBP (mmHg)	132.61 ± 14.14	130 (100–180)	134.03 ± 15.21	130 (100–180)	−0.10 (−0.28 to 0.08)
FIB-4	1.65 ± 0.47	1.61 (0.1–2.66)	5.2 ± 3.98	3.55 (2.67–25.26)	−0.89 (−1.08 to −0.70)
AST	24 ± 8.25	22 (1.2–74)	61.13 ± 52.09	38.5 (16–197)	**−0.71 (−0.90 to −0.53)**
ALT	23.81 ± 17.32	19 (7–221)	26.97 ± 25.52	16.5 (4–180)	−0.12 (−0.30 to 0.05)
GGT	26.61 ± 30.89	16 (5–158)	50.87 ± 47.54	28 (6–158)	**−0.51 (−0.69 to −0.33)**
FBG (mg/dl)	104 ± 23.32	99 (59–300)	103.42 ± 21.65	99 (80–229)	0.03 (−0.16 to 0.21)
HbA1c (mmol/mol)	39.79 ± 9.53	38 (19–128)	38.78 ± 7.87	38 (18–82)	0.13 (−0.06 to 0.32)
Total cholesterol (mg/dl)	187.92 ± 37.44	187 (79–386)	174.67 ± 32.97	175 (85–270)	**0.36 (0.18–0.55)**
Triglycerides (mg/dl)	105.32 ± 54.91	94 (28–520)	93.01 ± 52.43	75.5 (17–323)	**0.23 (0.04–0.41)**
Platelets (103 cells/mm^3^)	235.88 ± 54.6	231 (104–563)	186.69 ± 52.28	184 (64–333)	**0.91 (0.72–1.10)**

*N* = 731. All data are shown as mean ± SD, median (min to max) for continuous variables and as *n* (%) for proportions. Statistically significant data are indicated in bold type. *Cohen’s effect size or Glass’ delta effect size according to the homogeneity of variance for continuous variables and prevalence difference for proportions. BMI, body mass index; DBP, diastolic blood pressure; SBP, systolic blood pressure; AST, aspartate amino transferase; ALT, alanine amino transferase; GGT, gamma-glutamyl transferase; FBG, fasting blood glucose.

Table 3 shows the ophthalmological parameters in subjects with a FIB-4 below and above the 2.67 cut-off. Subjects with lower FIB-4 had higher thickness of GCC [SE 0.20 (0.02–0.38)] and higher VD both at superficial [SE 0.25 (0.07–0.43)] and deep [SE 0.31 (0.13–0.49)] level.

**TABLE 3:** Description of the whole sample at ophthalmological assessment.

	FIB < 2.67		FIB ≥ 2.67		
	Mean ± SD	Median (min to max)	Mean ± SD	Median (min to max)	Effect size*
Whole retina SVD	50.3 ± 4.05	50.9 (33.05–58.51)	49.27 ± 4.46	50.45 (36.77–57.39)	**0.25 (0.07–0.43)**
Whole retina DVD	54.86 ± 4.86	55.94 (29.51–63.29)	53.14 ± 5.62	54.35 (21.32–62.5)	**0.31 (0.13–0.49)**
GCC total Th (μm)	96.55 ± 13.4	94.86 (44.75–237.85)	93.82 ± 14.44	94.15 (58.03–166.51)	**0.20 (0.02–0.38)**
ONH RNFL Th (μm)	96.09 ± 10.63	97 (61–127)	94.94 ± 12.43	97 (57–128)	0.09 (−0.09 to 0.27)

*N* = 731. All data are shown as mean ± SD, median (min to max) for continuous variables and as *n* (%) for proportions. Statistically significant data are indicated in bold type. *Cohen’s effect size or Glass’ delta effect size according to the homogeneity of variance for continuous variables and prevalence difference for proportions. SVD, superficial vessel density; DVD, deep vessel density; GCC, ganglion cell complex; ONH, optic nerve head; RNFL, retinal nerve fiber layer.

**FIGURE 2:**
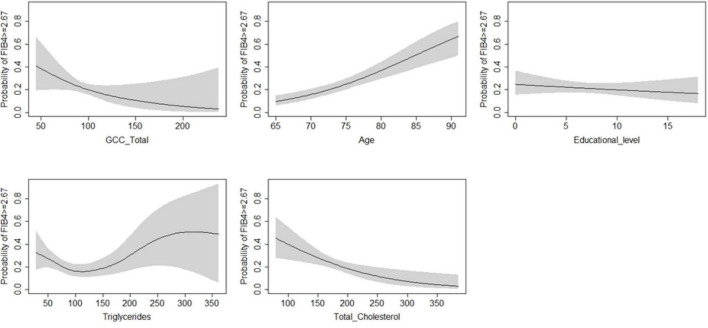
Generalized additive model on the probability of FIB-4 ≥ 2.67 (fibrosis/no fibrosis) as dependent variable and regressors.

According to the regression models shown in Table 4, each unit increase in whole retina superficial vessel density (SVD) (OR: 0.94, 95% CI: 0.90–0.98, SE: 0.02), and whole retina DVD (OR: 0.94, 95% CI: 0.91–0.97, SE: 0.01) was inversely associated with a higher risk of liver fibrosis (Table 4). However, these two associations lost their effect after adjustment. Moreover, the increase in GCC thickness was inversely associated with a FIB-4 score above the cut-off in both the raw model (OR: 0.98; 95% CI: 0.96–0.99; SE: 0.01) and after adjustment (OR: 0.98; 95% CI: 0.97–0.99; SE: 0.01) (Table 3). To assess important non-linear relations between the associated variable of interest (GCC) and the fibrosis risk, we performed a generalized additive model (GAM). The results of GAM (Table 5) showed no non-linear relationship between GCC and the risk score [estimate degrees of freedom (edf) = 1.18]. Only the triglycerides had some significant non-linear relationship (edf = 3.2). Moreover, relative probability functions for each GAM relation are shown in Figure 2: only triglycerides showed a complex non-linear association with the probability of FIB score ≥2.67.

**TABLE 4:** Logistic regression models on the FIB-4 cut-off of >2.67 (fibrosis/no fibrosis) and regressors.

	Raw model				Adjusted model			
	OR	CI 95%	SE	*P*-value	OR	CI 95%	SE	*P*-value
Whole retina SVD	**0.94**	**0.90–0.98**	**0.02**	**<0.01**	0.99	0.94–1.05	0.02	0.85
Age (years)					1.19	1.10–1.16	0.02	<0.01
Sex (female)					0.73	0.46–1.18	0.24	0.20
Education (years)					0.98	0.92–1.05	0.03	0.69
Hypertension (yes)					0.89	0.48–1.68	0.32	0.73
Diabetes (yes)					0.74	0.34–1.63	0.40	0.62
Alcohol consumption					1.78	0.66–4.80	0.50	0.25
Triglycerides (mg/dl)					1.01	0.99–1.01	0.02	0.94
Cholesterol (mg/dl)					0.98	0.98–0.99	0.03	<0.01
Whole retina DVD	**0.94**	**0.91–0.97**	**0.01**	**<0.01**	1.01	0.96–1.06	0.02	0.85
Age (years)					1.12	1.10–1.17	0.02	<0.01
Sex (female)					0.72	0.45–1.17	0.24	0.20
Education (years)					0.98	0.92–1.05	0.03	0.69
Hypertension (yes)					0.88	0.47–1.66	0.32	0.73
Diabetes (yes)					0.75	0.34–1.65	0.40	0.62
Alcohol consumption					1.76	0.66–4.75	0.50	0.25
Triglycerides (mg/dl)					0.99	0.99–1.01	0.01	0.94
Cholesterol (mg/dl)					0.9 8	0.98–0.99	0.01	<0.01
GCC total Th	**0.98**	**0.96–0.99**	**0.01**	**0.02**	**0.98**	**0.97–0.99**	**0.01**	**0.04**
Age (years)					1.11	1.11–1.16	0.02	<0.01
Sex (female)					0.72	0.45–1.18	0.24	0.19
Education (years)					0.98	0.91–1.05	0.03	0.61
Hypertension (yes)					0.80	0.43–1.52	0.32	0.51
Diabetes (yes)					0.71	0.32–1.58	0.40	0.57
Alcohol consumption					1.72	0.64–4.67	0.50	0.28
Triglycerides (mg/dl)					1.01	0.98–1.01	0.02	0.95
Cholesterol (mg/dl)					0.98	0.97–0.99	0.03	<0.01
ONH RNFL Th	0.99	0.97–1.01	0.01	0.26	0.98	0.97–1.01	0.01	0.34
Age (years)					1.11	1.10–1.16	0.02	<0.01
Sex (female)					0.73	0.46–1.19	0.24	0.21
Education (years)					0.98	0.92–1.05	0.03	0.68
Hypertension (yes)					0.86	0.46–1.63	0.32	0.66
Diabetes (yes)					0.73	0.33–1.61	0.40	0.60
Alcohol consumption					1.77	0.66–4.77	0.50	0.26
Triglycerides (mg/dl)					1.01	1.01–1.01	0.01	0.98
Cholesterol (mg/dl)					0.99	0.98–0.99	0.01	<0.01

Statistically significant data are indicated in bold type. OR, odds ratio; CI, confidence interval; SE, standard error; Th, thickness; SVD, superficial vessel density; DVD, deep vessel density; GCC, ganglion cell complex; ONH, optic nerve head; RNFL, retinal nerve fiber layer.

**TABLE 5:** Generalized additive model regression on the FIB-4 cut-off of ≥2.67 (fibrosis/no fibrosis) as dependent variable and regressors.

	edf	Reference of degree of freedom	Chi-square	*P*-value
GCC total Th (μm)	1.18	1.35	3.09	0.09
Age (years)	1.00	1.00	30.25	<0.01
Educational_level (years)	1.00	1.00	0.59	0.44
Triglycerides (mg/dl)	3.20	3.99	9.18	0.06
Total_cholesterol (mg/dl)	1.00	1.00	8.46	<0.01

GCC, gangliar cellular complex.

Supplementary Table 1 shows a sensitivity analysis performed in order to underline any significant modification in coefficients. No significant variation in OR were found.

## Discussion

This study investigated cross-sectional associations between the risk of liver fibrosis and retinal OCT-A features, particularly neuroretinal thickness and vascular density, in a cohort of older adults. A major finding was neuroretinal thinning in subjects with FIB-4 ≥ 2.67, mainly involving the GCC, with an inverse association between the GCC thickness and liver score, also maintained after adjusting for possible confounders such as age, sex, education, hypertension, diabetes mellitus, and alcohol consumption. Using the GAM, we demonstrated that this adjusted association was explained by a completely linear function. Hence, our results support the thesis that there could be a close link between liver metabolic deficiency and retinal neurodegeneration.

Our findings extend previous research by establishing the impact of liver fibrosis on retinal features in an older population. We applied a liver fibrosis score that can be computed from normal laboratory testing and has been validated across a wide range of underlying liver diseases. Our findings are conceptually consistent with the growing knowledge that liver fibrosis has repercussions on human biology and disease regardless of the underlying etiology, as has been found, for example, in cerebrovascular disease and heart disease (Seo et al., 2016; Ostovaneh et al., 2018). Moreover, our recent analysis performed in the same population highlighted a positive association between the risk of NAFLD and dementia (Lampignano et al., 2021).

Retinal ganglion cells are characterized by specific common features, such as the location of their somata in the GCL, the arborization of their dendrites in the IPL, and the gathering of their axons in the RNFL, which, by projecting to the lateral geniculate nucleus, connect their glutamatergic synapses with the second-order neurons of the visual pathway (Shahlaee et al., 2016). All three layers together compose the GCC. Retinal ganglion cells and RNFL thinning identifying a neuroretinal degeneration has been associated with different systemic conditions, including neurological disorders, respiratory disorders, psychiatric disease, autoimmune disease, inflammatory conditions, malignancy, infectious diseases, vitamin deficiency, and side effects of medications (Mukherjee et al., 2019).

With a protein synthesis rate double that of skeletal muscle, the retina is one of the most metabolically active tissues (Fort et al., 2014), like the liver parenchyma. This high basal anabolic activity corresponds to retinal insulin receptor and Akt kinase activities, which are double those of skeletal muscle and comparable to those of the post-prandial liver and remain constant during feeding and fasting (Reiter et al., 2003). The kinase, the mechanistic target of rapamycin (mTOR), is at the heart of a critical signaling hub that regulates fundamental cellular functions. When dysregulated, it can lead to disorders such as diabetes and liver disease, as well as cancer and neurological diseases (Saxton and Sabatini, 2017; Wang et al., 2019). Moreover, in a series of studies, mTOR has been shown to play a crucial role in retinal development (Choi et al., 2018) and axonal survival after optic nerve injury (Qiao and Duan, 2015; Morgan-Warren et al., 2016).

A potential link between liver fibrosis and neuroretinal degeneration could be the liver X receptor (LXR) function. LXRs play a crucial role in lipid metabolism, glucose homeostasis, inflammation (Courtney and Landreth, 2016; Mutemberezi et al., 2016), and CNS functions (Cai et al., 2018; Song et al., 2019). They also play an important role in susceptibility to liver fibrosis (Beaven et al., 2011; O’Mahony et al., 2015). These receptors have been detected in human retinal ganglion cells (Hammer et al., 2017) and the retinal pigment epithelium-choroid complex (Song et al., 2019; Choudhary et al., 2020). They have been reported to protect against retinal degeneration (Hammer et al., 2017), so as their expression decreases with age, the risk for chronic retinal pathologies increases (Song et al., 2019; Choudhary et al., 2020). Recently, Song et al. confirmed that LXR is expressed in the retina and optic nerve and their loss leads to the loss of ganglion cells.

Damage of the neuroretinal elements but also of retinal microvascular components was observed in diabetes (Gardner and Davila, 2017) and chronic kidney disease (Peng et al., 2020). Previous papers reported that liver disease as NAFLD is a risk factor for retinal artery lesions, leading to arteriovenous compression, narrowing, and irregularity of retinal arterioles (Josef et al., 2013; Yang et al., 2015). The mechanisms underlying the association between retinal artery lesions and NAFLD might include endothelial dysfunction, oxidative stress, inflammation, inflammatory cytokines, dyslipidemia, and glucose metabolism disorders (Targher et al., 2004).

We did not observe any associations between retinal vascular findings and a higher risk of liver fibrosis. Some considerations should be made. This could result from the fact that FIB is a probability score, thus a non-deterministic stratification of subjects with a high probability of fibrosis. Moreover, the score was validated on liver fibrosis in a population aged 65+ years, showing a higher specificity (85%) than sensitivity (53%) (McPherson et al., 2017). Therefore, this score could perform better for detecting truly negative healthy subjects but with a likelihood of having deranged liver biomarkers than a pre-clinical stage of fibrosis.

### Strength and limitations

The strengths of the present study include its large population-based sample size and the generalizability of the results to southern Mediterranean older populations.

Besides, this study is the first to evaluate more specific quantitative parameters of retinal vasculature, as compared to previous studies where morphologic parameters were evaluated using only direct fundoscopy which could be influenced by inter-examiner variability.

Moreover, OCT scanning was combined with an ophthalmological clinical examination to avoid optical interferences due to ocular media abnormalities. Furthermore, we considered the measurements of one eye randomly for each subject as a good practice for statistical analysis (Armstrong, 2013), although, in all age groups a moderate degree of interocular asymmetry in retinal layer thickness, including GCC and RNFL (Yang et al., 2016), and retinal vascular features (Cameron et al., 2017) has previously been reported. Moreover, OCT-A also investigates abnormalities in terms of vessel perfusion, and has been shown to be a high-performance “*in vivo* model” for neural and microvascular tissue degeneration (Bulut et al., 2018; Zhang et al., 2020). However, information on liver fibrosis status, as detected by transient elastography (FibroScan) or biopsy, was unavailable. To address this limitation, we used the FIB-4 score as a surrogate, as this is a more accessible screening tool for physicians, especially general practitioners, and was shown to predict important health outcomes in the general population, independent of the presence of liver disease (So-Armah et al., 2020; Schonmann et al., 2021; Higashiura et al., 2022) Lastly, even though there is evidence of the association between smoking and liver diseases (Rutledge and Asgharpour, 2020) and retinal abnormalities (Cho et al., 2014), the smoking status could not be used as a covariate in this study since there are no data on lifetime smoking habits.

## Conclusion

In conclusion, although it is widely known that severe liver disease is typically associated with cognitive decline, our results add to the current evidence that the presence of liver fibrosis might be an essential risk factor for retinal neurodegeneration. This finding raises several additional considerations related to the possibility of using OCT-A to follow the trajectories of retinal abnormalities and define precise thresholds that can predict and classify liver disease. Moreover, it could bring interesting novelties to the physiopathological mechanisms underlying the strong association between deranged liver biomarkers and cognitive impairment. Since this study does not allow an exploration of causal inferences between liver and neuroretinal thinning, the topic warrants further research, particularly as regards the longitudinal evaluation of retinal features in patients within the “grey area,” i.e., with a FIB-4 score not allowing to rule out or detect liver fibrosis.

## Members of the Eye Clinic Research Group

Department of Basic Medical Sciences, Neuroscience and Sense Organs, University of Bari “Aldo Moro”, Bari, Italy: Giulia Maria Pia Bisceglia, Rosa Buonamassa, Flavio Cassano, Arcangelo Clemente, Pierfrancesco Digregorio, Roberta Galati, Marida Gaudiomonte, Antonella Guglielmi, Luca Landini, Francesca Palumbo, Pasquale Pasculli, Giovanni Petruzzella, Michele Santoro, Giacomo Scotti, and Roberto Semeraro.

## Data availability statement

The raw data supporting the conclusions of this article will be made available by the authors, without undue reservation.

## Ethics statement

The studies involving human participants were reviewed and approved by IRB of the National Institute of Gastroenterology “S. De Bellis,” protocol no. 68 CE De Bellis. The patients/participants provided their written informed consent to participate in this study.

## Author contributions

RS, LL, and AN: conceptualization. RS: methodology, writing—review and editing, project administration, and funding acquisition. FC: formal analysis and data curation. Eye Clinic Research Group, LL, AN, IB, RZ, ST, RT, CG, SD, and AS: investigation. LL and AN: writing—original draft preparation. CC, GD, GS, FB, and GA: supervision. All authors read and agreed to the published version of the manuscript.

## Footnotes

1
http://www.stard-statement.org/

2
https://www.strobe-statement.org/
