# Supplementary material for: Liver fibrosis and retinal features in an older Mediterranean population: Results from the Salus in Apulia study
Source: Front Neurosci. 2022 Dec 15;16:1048375. doi: 10.3389/fnins.2022.1048375 (PMC9798127; doi:10.3389/fnins.2022.1048375)
Supplement: Supplementary file 1 [file Table_1.DOCX]

Table S1 _ Sensitivity analysis

|  | OR | CI 95% | SE | p value | OR | CI 95% | SE | p value | OR | CI 95% | SE | p value | OR | CI 95% | SE | p value |
| --- | --- | --- | --- | --- | --- | --- | --- | --- | --- | --- | --- | --- | --- | --- | --- | --- |
| Whole retina SVD | **0.94** | **0.91 to 0.98** | **0.02** | **<0.01** | 0.97 | 0.92 to 1.01 | 0.02 | 0.24 | 0.97 | 0.93 to 1.02 | 0.02 | 0.34 | 0.97 | 0.92 to 1.02 | 0.02 | 0.30 |
| Whole retina DVD |  |  |  |  | **0.95** | **0.91 to 0.98** | **0.01** | **<0.01** | **0.95** | **0.91 to 0.99** | **0.01** | **0.01** | **0.95** | **0.91 to 0.99** | **0.01** | **0.01** |
| GCC Total Th |  |  |  |  |  |  |  |  | 0.98 | 0.97 to 1.01 | 0.01 | 0.12 | 0.98 | 0.96 to 1.01 | 0.01 | 0.11 |
| ONH RNFL Th |  |  |  |  |  |  |  |  |  |  |  |  | 1.01 | 0.98 to 1.02 | 0.01 | 0.53 |
| Age (years) |  |  |  |  |  |  |  |  |  |  |  |  |  |  |  |  |
| Sex (Female) |  |  |  |  |  |  |  |  |  |  |  |  |  |  |  |  |
| Education (years) |  |  |  |  |  |  |  |  |  |  |  |  |  |  |  |  |
| Hypertension (yes) |  |  |  |  |  |  |  |  |  |  |  |  |  |  |  |  |
| Diabetes (yes) |  |  |  |  |  |  |  |  |  |  |  |  |  |  |  |  |
| Alcohol consumption |  |  |  |  |  |  |  |  |  |  |  |  |  |  |  |  |
| Triglycerides (mg/dl) |  |  |  |  |  |  |  |  |  |  |  |  |  |  |  |  |
| Cholesterol (mg/dl) |  |  |  |  |  |  |  |  |  |  |  |  |  |  |  |  |
|  | OR | CI 95% | SE | p value | OR | CI 95% | SE | p value | OR | CI 95% | SE | p value | OR | CI 95% | SE | p value |
| Whole retina SVD | 0.99 | 0.94 to 1.05 | 0.02 | 0.93 | 0.99 | 0.94 to 1.05 | 0.02 | 0.93 | 0.99 | 0.94 to 1.05 | 0.02 | 0.92 | 0.99 | 0.94 to 1.05 | 0.02 | 0.95 |
| Whole retina DVD | 0.97 | 0.93 to 1.01 | 0.02 | 0.20 | 0.97 | 0.93 to 1.01 | 0.02 | 0.20 | 0.97 | 0.93 to 1.01 | 0.02 | 0.19 | 0.97 | 0.93 to 1.01 | 0.02 | 0.21 |
| GCC Total Th | 0.98 | 0.96 to 1.01 | 0.01 | 0.06 | 0.98 | 0.96 to 1.01 | 0.01 | 0.06 | 0.98 | 0.96 to 1.01 | 0.01 | 0.06 | 0.98 | 0.96 to 0.99 | 0.01 | 0.04 |
| ONH RNFL Th | 1.01 | 0.99 to 1.03 | 0.01 | 0.21 | 1.01 | 0.99 to 1.03 | 0.01 | 0.20 | 1.01 | 0.92 to 1.03 | 0.01 | 0.19 | 1.01 | 0.99 to 1.03 | 0.01 | 0.19 |
| Age (years) | **1.10** | **1.07 to 1.14** | **0.01** | **<0.01** | **1.10** | **1.07 to 1.14** | **0.01** | **<0.01** | **1.09** | **1.06 to 1.13** | **0.01** | **<0.01** | **1.10** | **1.06 to 1.13** | **0.01** | **<0.01** |
| Sex (Female) |  |  |  |  | 0.91 | 0.61 to 1.35 | 0.20 | 0.65 | 0.90 | 0.60 to 1.34 | 0.20 | 0.60 | 0.90 | 0.60 to 1.35 | 0.20 | 0.63 |
| Education (years) |  |  |  |  |  |  |  |  | 0.95 | 0.90 to 1.01 | 0.02 | 0.11 | 0.95 | 0.90 to 1.01 | 0.02 | 0.12 |
| Hypertension (yes) |  |  |  |  |  |  |  |  |  |  |  |  | 0.74 | 0.44 to 1.24 | 0.26 | 0.25 |
| Diabetes (yes) |  |  |  |  |  |  |  |  |  |  |  |  |  |  |  |  |
| Alcohol consumption |  |  |  |  |  |  |  |  |  |  |  |  |  |  |  |  |
| Triglycerides (mg/dl) |  |  |  |  |  |  |  |  |  |  |  |  |  |  |  |  |
| Cholesterol (mg/dl) |  |  |  |  |  |  |  |  |  |  |  |  |  |  |  |  |
|  | OR | CI 95% | SE | p value | OR | CI 95% | SE | p value | OR | CI 95% | SE | p value | OR | CI 95% | SE | p value |
| Whole retina SVD | 0.99 | 0.94 to 1.05 | 0.02 | 0.93 | 0.99 | 0.93 to 1.05 | 0.03 | 0.81 | 0.99 | 0.93 to 1.05 | 0.03 | 0.84 | 0.99 | 0.93 to 1.06 | 0.01 | 0.89 |
| Whole retina DVD | 0.97 | 0.93 to 1.01 | 0.02 | 0.20 | 1.01 | 0.96 to 1.07 | 0.02 | 0.53 | 1.01 | 0.96 to 1.07 | 0.02 | 0.53 | 1.02 | 0.96 to 1.07 | 0.01 | 0.48 |
| GCC Total Th | 0.98 | 0.95 to 0.99 | 0.01 | 0.04 | 0.98 | 0.96 to 1.01 | 0.01 | 0.15 | 0.98 | 0.96 to 1.01 | 0.01 | 0.16 | 0.98 | 0.96 to 1.01 | 0.01 | 0.12 |
| ONH RNFL Th | 1.01 | 0.99 to 1.03 | 0.01 | 0.20 | 0.99 | 0.97 to 1.02 | 0.01 | 0.78 | 0.99 | 0.97 to 1.02 | 0.01 | 0.82 | 1.01 | 0.96 to 1.02 | 0.01 | 0.98 |
| Age (years) | **1.10** | **1.06 to 1.13** | **0.01** | **<0.01** | **1.12** | **1.07 to 1.17** | **0.02** | **<0.01** | **1.12** | **1.07 to 1.17** | **0.02** | **<0.01** | **1.12** | **1.07 to 1.73** | **0.01** | **<0.01** |
| Sex (Female) | 0.90 | 0.60 to 1.34 | 0.20 | 0.62 | 0.69 | 0.43 to 1.12 | 0.25 | 0.14 | 0.69 | 0.43 to 1.12 | 0.24 | 0.14 | 0.71 | 0.44 to 1.16 | 0.24 | 0.17 |
| Education (years) | 0.95 | 0.90 to 1.01 | 0.02 | 0.11 | 0.98 | 0.93 to 1.05 | 0.03 | 0.72 | 0.98 | 0.92 to 1.05 | 0.03 | 0.71 | 0.98 | 0.91 to 1.05 | 0.01 | 0.59 |
| Hypertension (yes) | 0.74 | 0.44 to 1.25 | 0.26 | 0.27 | 0.77 | 0.41 to 1.46 | 0.32 | 0.43 | 0.78 | 0.41 to 1.46 | 0.32 | 0.44 | 0.79 | 0.42 to 1.51 | 0.32 | 0.48 |
| Diabetes (yes) | 0.79 | 0.44 to 1.43 | 0.29 | 0.44 | 0.98 | 0.50 to 1.91 | 0.33 | 0.95 | 1.01 | 0.50 to 1.91 | 0.34 | 0.96 | 0.82 | 0.41 to 1.65 | 0.35 | 0.59 |
| Alcohol consumption |  |  |  |  | 1.64 | 0.51 to 4.35 | 0.49 | 0.32 | 1.63 | 0.61 to 4.32 | 0.49 | 0.33 | 1.70 | 0.53 to 4.59 | 0.50 | 0.29 |
| Triglycerides (mg/dl) |  |  |  |  |  |  |  |  | 0.99 | 0.93 to 1.01 | 0.01 | 0.51 | 1.01 | 0.99 to 1.02 | 0.01 | 0.97 |
| Cholesterol (mg/dl) |  |  |  |  |  |  |  |  |  |  |  |  | **0.98** | **0.98 to 0.99** | **0.01** | **<0.01** |
